# Development of Custom Wall-Less Cardiovascular Flow Phantoms with Tissue-Mimicking Gel

**DOI:** 10.1007/s13239-021-00546-7

**Published:** 2021-06-02

**Authors:** Megan E. Laughlin, Sam E. Stephens, Jamie A. Hestekin, Morten O. Jensen

**Affiliations:** 1 Department of Biomedical Engineering, University of Arkansas, John A. White Jr. Engineering Hall, 790 W. Dickson St. #120, Fayetteville, AR 72701 USA; 2 Department of Chemical Engineering, University of Arkansas, 3202 Bell Engineering Center, Fayetteville, AR 72701 USA

**Keywords:** Flow phantoms, *In vitro*, Tissue simulation, 3D printing, Polyvinyl alcohol, Blood vessels, Medical device testing

## Abstract

**Purpose:**

Flow phantoms are used in experimental settings to aid in the simulation of blood flow. Custom geometries are available, but current phantom materials present issues with degradability and/or mimicking the mechanical properties of human tissue. In this study, a method of fabricating custom wall-less flow phantoms from a tissue-mimicking gel using 3D printed inserts is developed.

**Methods:**

A 3D blood vessel geometry example of a bifurcated artery model was 3D printed in polyvinyl alcohol, embedded in tissue-mimicking gel, and subsequently dissolved to create a phantom. Uniaxial compression testing was performed to determine the Young’s moduli of the five gel types. Angle-independent, ultrasound-based imaging modalities, Vector Flow Imaging (VFI) and Blood Speckle Imaging (BSI), were utilized for flow visualization of a straight channel phantom.

**Results:**

A wall-less phantom of the bifurcated artery was fabricated with minimal bubbles and continuous flow demonstrated. Additionally, flow was visualized through a straight channel phantom by VFI and BSI. The available gel types are suitable for mimicking a variety of tissue types, including cardiac tissue and blood vessels.

**Conclusion:**

Custom, tissue-mimicking flow phantoms can be fabricated using the developed methodology and have potential for use in a variety of applications, including ultrasound-based imaging methods. This is the first reported use of BSI with an *in vitro* flow phantom.

## Introduction

Flow phantoms serve as test beds that simulate blood flow for experimental studies. In particular, they are often used in applications utilizing various imaging modalities, testing medical devices, and alongside computational models.^11^, ^23^, ^25^, ^58^ The high-cost of commercial phantoms ($500 to several thousand) is a major limitation, while other established materials and methods for phantom fabrication present issues with degradability and achieving complex, custom geometries.^15^, ^38^, ^39^ 3D printing has offered a low-cost option of creating custom phantoms, including patient-specific.^9^, ^11^, ^19^, ^24^, ^51^, ^53^, ^54^ Multiple studies have created phantoms by embedding a 3D print in various materials with subsequent removal to yield the desired geometry, most often producing wall-less phantoms, consisting of a tissue-mimicking block with a void or hollow lumen, as opposed to a walled phantom that has a thin, variable material thickness.^2^, ^23^, ^39^, ^40^, ^48^, ^49^, ^60^ These wall-less phantoms are particularly useful in ultrasound-based imaging applications; however, the mechanical properties of the materials used are often not prioritized and thus their mimicry of human tissue in this regard is limited.

An oil-based, synthetic tissue-mimicking gel is available commercially from Humimic Medical, LLC (Greenville, SC) in five different clear gel types (Gel 0–4), each with a different stiffness. This gel has been recognized widely, and used for example by military and law enforcement in the US and internationally, as well as in TV shows such as Mythbusters, to mimic human tissue. Recommended methods of use have been established by the manufacturer where the gel can easily and relatively quickly be melted in a standard oven with minimal oversight. The cooling/curing process is also simple and time efficient with a full phantom taking a few hours, depending on size, with no additional steps. The gel can be reheated up to ten times while maintaining its original properties.^26^ Since the source materials are 100% synthetic, the gel can be stored at room temperature indefinitely with no required additives.

A current issue with previous methods for fabricating phantoms using the embedment and subsequent removal of 3D printed inserts is that many 3D printable materials require harsh chemicals, such as acetone, limonene, or chlorinated solvents, for their dissolution that are not compatible for use with the gel. Removal of these materials using heat is precluded by the fact that their melting temperatures are often higher than that of the gel. Polyvinyl alcohol (PVA) is a synthetic polymer often used as a support material in 3D printing due to its water solubility, making it an optimal alternative. Thus, the purpose of this study is to develop a method of fabricating custom wall-less flow phantoms from tissue-mimicking gel using 3D printed PVA inserts and demonstrate flow visualization of the phantom using ultrasound-based imaging modalities. While this gel is currently being used for phantom fabrication, the specific gel types have yet to be characterized to ensure the appropriate gel is being used to simulate a given tissue. Therefore, we also aim to determine the Young’s moduli of the available gel types and identify tissues that phantoms of this material may be suitable for mimicking, specifically types of cardiovascular tissue.

## Materials and Methods

### Phantom Fabrication

**Figure 1:**
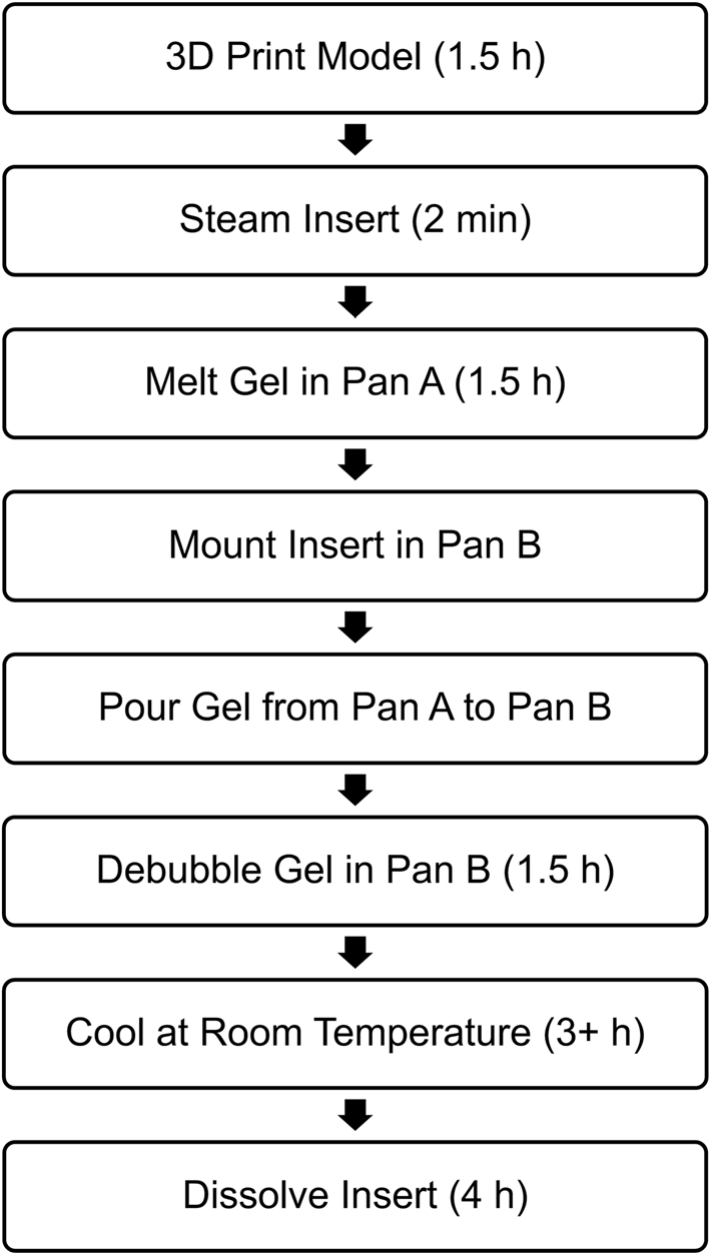
Flowchart of the fabrication process, including the time required for each step, for custom wall-less flow phantoms from tissue-mimicking gel using 3D printed PVA inserts. Three hours is sufficient for gel cooling; however, the gel was typically allowed to cool for 24 h.

**Figure 2:**
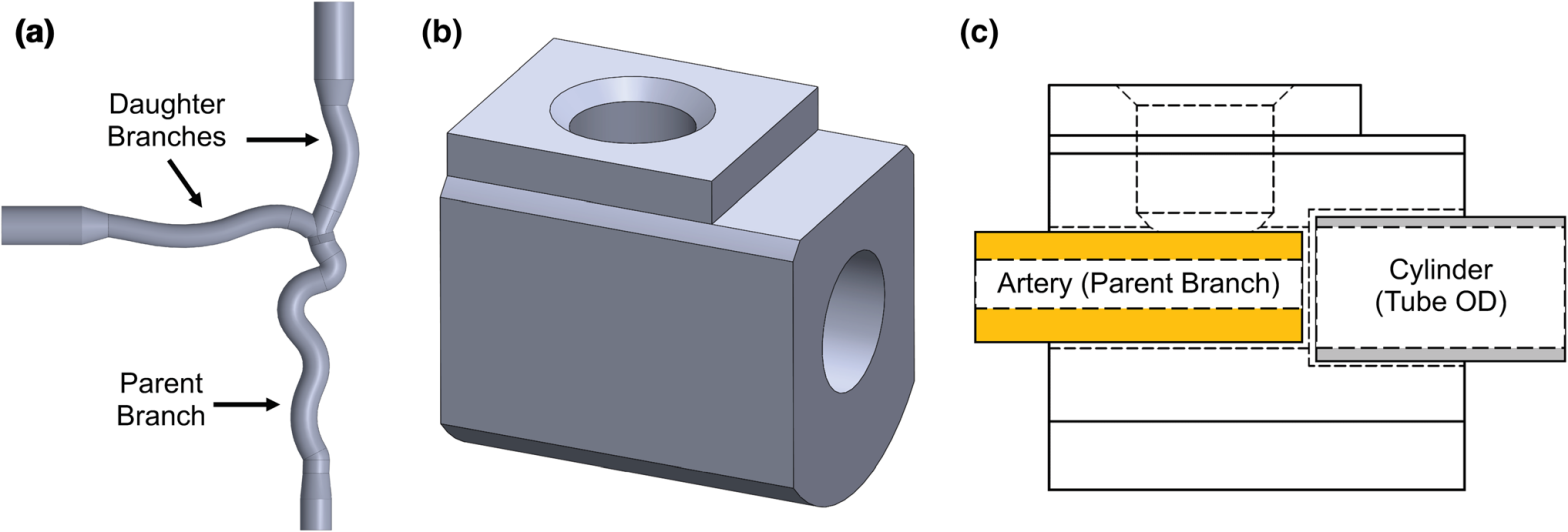
SolidWorks renderings of the (a) bifurcated artery and (b) Luer anchor; (c) Schematic of how the bifurcated artery parent branch and cylinder are fit with the Luer anchor. In the completed phantom, tubing will be inserted where the cylinder is denoted and a smooth transition of flow from tubing to conduit will be allowed.

A flowchart of the phantom fabrication process is given in Fig. 1. A bifurcated artery model containing a small channel through it was created in SolidWorks (Dassault Systemes, VeÂlizy-Villacoublay, France) based on the tortuosity and mean diameters of the middle cerebral, internal carotid, and basilar arteries (Fig. 2a).^45^, ^52^, ^55^ The ends of each daughter branch were gradually tapered such that the outer diameter was equal to the inner diameter of the tubing that would be connected to the phantom for providing fluid flow. Additionally, a cylinder model also containing a small channel was created with outer diameter corresponding that of the tubing. Two types of anchors to be used for securing tubing into the phantom were created: simple and Luer. For the simple anchor, a disk was cut from a polyvinyl chloride rod and a hole with the diameter of the bifurcated artery daughter branches was drilled through its center. The Luer anchor was created in SolidWorks as a block with two concentric holes of differing diameter and a top hole (Fig. 2b). The concentric holes had one diameter corresponding to that of the bifurcated artery parent branch and the other of the cylinder, while the top hole was sized for a screw-in Luer fitting. A schematic of how the bifurcated artery parent branch and cylinder are sized to fit the Luer anchor to provide a smooth transition of flow within the phantom is given in Fig. 2c. All SolidWorks models were saved as .STL files for subsequent 3D printing.

#### 3D Printing

**Figure 3:**
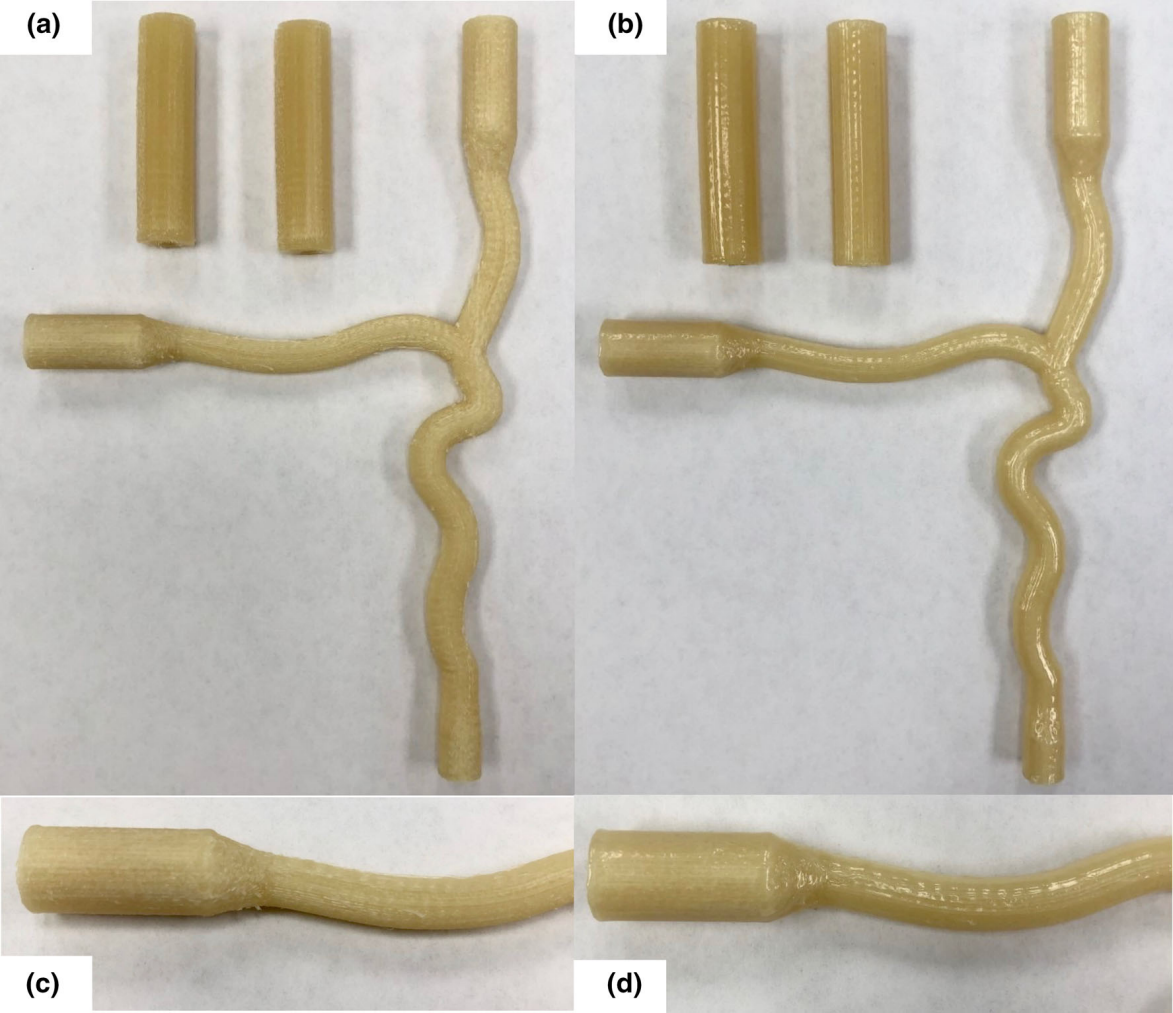
The effects of steaming on the 3D printed PVA inserts. (a) Prior to steaming, ridges are present on the surface, which is common with extrusion printing using filament; (b) A smooth, uniform surface with a glossy appearance is achieved by steaming the inserts; Close-up of surface (c) before and (d) after steaming.

The bifurcated artery and cylinder models were printed using PVA on a Makerbot Replicator 2X (Makerbot, Brooklyn, NY) extrusion 3D printer with blue painter’s tape as the print surface. Extruder and print bed temperatures were 185 and 60 °C, respectively. Additional print settings included a raft to model spacing of 0.7 mm, 100% infill, and relatively slow print speeds of 10 mm/s for the raft base, 20 mm/s for the first layer, and 50 mm/s for outlines, insets, and infill. The PVA prints, to be used as “inserts” and called such from this point on, were steamed in order to minimize surface roughness and bubble formation in the gel (Fig. 3). This was done by inserting a wire into the conduit of the insert, to hold the insert and be used as a handle. Then the insert was held above a continuous steam source (GS-8250, GOODSKY, Taichung City, Taiwan) while rotating to ensure all surfaces were smoothened. Whether the surface was sufficiently smooth was determined visually by if the entire surface displayed a glossy appearance with no noticeable ridges or lines still present. The steamed insert was suspended by the wire to fully dry before subsequent use or storage. PVA filament and inserts were stored in sealed containers alongside desiccant packets until ready for use due to the material’s susceptibility to moisture absorption. The Luer anchor was printed with a FormLabs high temperature resin (FLHTAM02) on a Form 2 stereolithography (SLA) 3D printer (FormLabs, Somerville, MA). Threads for the Luer fitting were tapped manually following printing and cleaning.

#### Embedding of 3D Printed Insert in Tissue-Mimicking Gel

**Figure 4:**
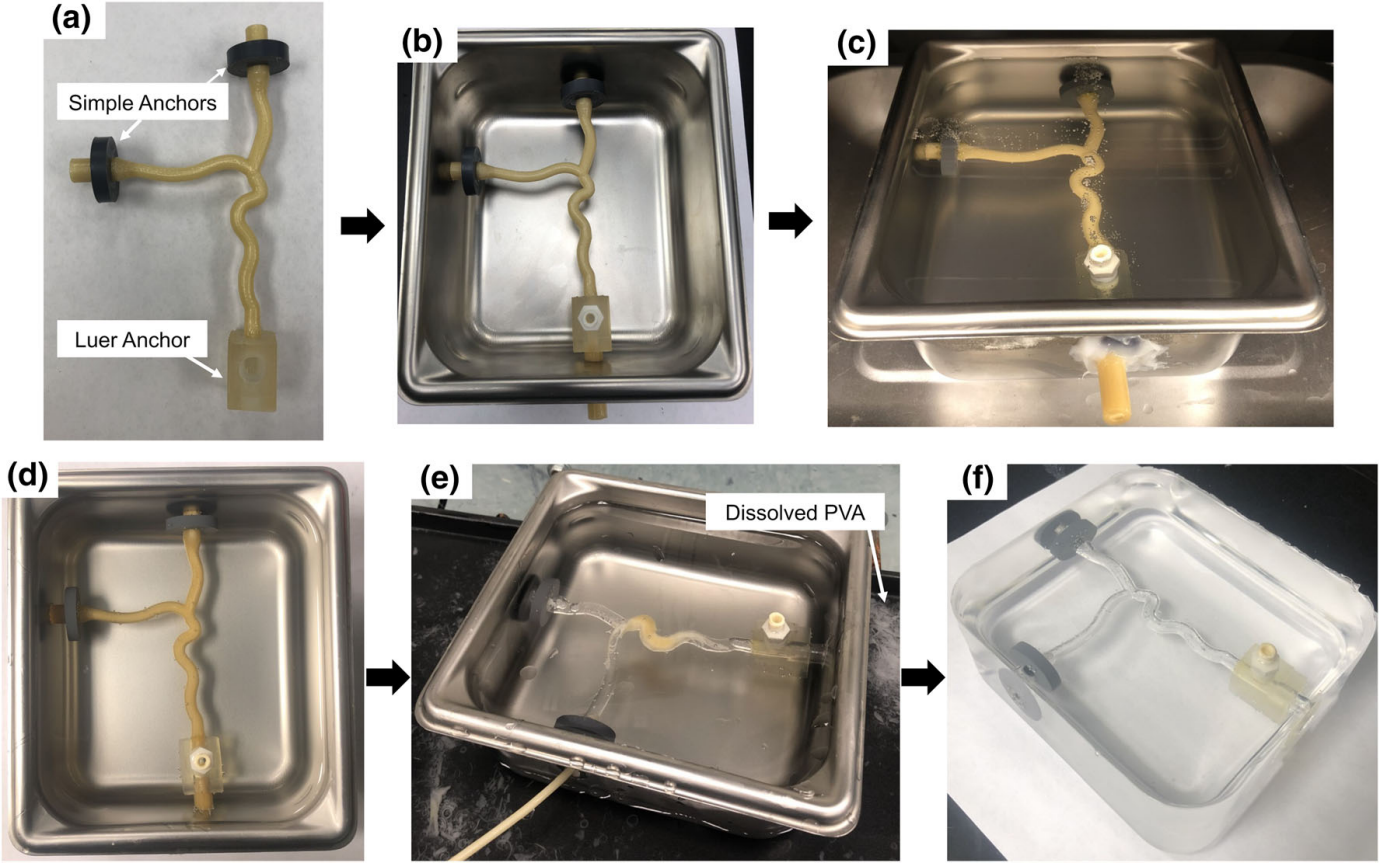
Phantom fabrication process. (a) Bifurcated artery insert with the simple and Luer anchors attached to the daughter and parent branches, respectively; (b) Anchored insert mounted in Pan B; (c) Pan B containing melted gel poured from Pan A in the oven to debubble. Bubbles form on the insert and can be seen rising to the gel surface; (d) Gel free of bubbles removed from the oven and allowed to cool at room temperature; (e) Dissolution of insert with warm water. Dissolved PVA is depicted by the arrow; (f) Completed phantom following removal from the pan.

Pieces of Gel 0 were placed in a stainless-steel pan (Pan A) and melted in an oven at 120 °C until no bubbles were present. Simple anchors were glued onto the daughter branches of the bifurcated artery insert with cyanoacrylate (CA) glue, while the ends of the parent branch and cylinder insert were glued into the Luer anchor (Fig. 4a). Holes were drilled in a separate stainless-steel pan (Pan B) to mount the anchored inserts (Fig. 4b). Once mounted, silicone was used to create a seal between the inserts and pan to ensure no gel would leak. The melted gel in Pan A was then slowly poured into Pan B, taking care to pour as far away from the insert as possible to minimize the formation of bubbles due to entrained air. After the gel was poured, Pan B was transferred into an oven at 120 °C to allow the gel to debubble (Fig. 4c). Once bubbles were no longer consistently rising from the insert surface and the gel as a whole had practically no bubbles present, as determined visually, Pan B was removed from the oven and left to cool at room temperature for 24 h (Fig. 4d). Additional bubbles that may have risen to or near the surface during the cooling process were extracted using a heat gun.

#### Dissolution of Insert

Warm water between 32–37 ºC was continuously pumped through the channel of the PVA insert using a pump connected to tubing that was directed at the inlet and/or outlets (Fig. 4e). In order to speed up the dissolution process, water was replaced as it cooled and/or became saturated with dissolved PVA. Once the insert was completely dissolved, the gel was carefully removed from the pan resulting in the completed phantom (Fig. 4f). Fingerprints and surface imperfections were removed by “heat polishing”, in which a heat gun was aimed at the surface approximately 3 to 5 inches away until the phantom was smoothed out and regained its clarity. To demonstrate flow capabilities, tubing was inserted into the phantom and connected to a pump within a fluid reservoir that provided continuous, steady flow to the phantom at 4 L/min. A small amount of CA glue, evenly spread across the circumference of the end of the tubing, firmly bonded the tube to the embedded anchors, providing a secure, leak-proof joint. The fluid consisted of water with red food coloring in order to easily see flow through the system.

### Mechanical Testing

Previously prepared disk-shaped samples (diameter = 39 ± 1.25 mm, height = 15.55 ± 0.45 mm) of the five gel types (*n* = 10) were obtained from Humimic Medical with an average diameter to height ratio of 2.53 ± 0.28. Uniaxial compression testing was performed at 21.5 ± 1 °C using an Instron 3300 mechanical testing system (Instron, Norwood, MA) by applying force perpendicular to the upper gel surface with a load rate of 20 N/min until 95% strain was reached. No preconditioning was required due to the synthetic nature of the material. Compressive stress–strain curves were obtained for all samples, and the Young’s modulus was determined by applying a linear fit to both the 0–25% and 25–75% strain portions of the curve to assess the mechanical behavior under different compressive strain ranges required for various applications. Additionally, *R*^2^ values were calculated to evaluate linearity and thus the goodness of fit achieved.

### Flow Visualization

**Figure 5:**
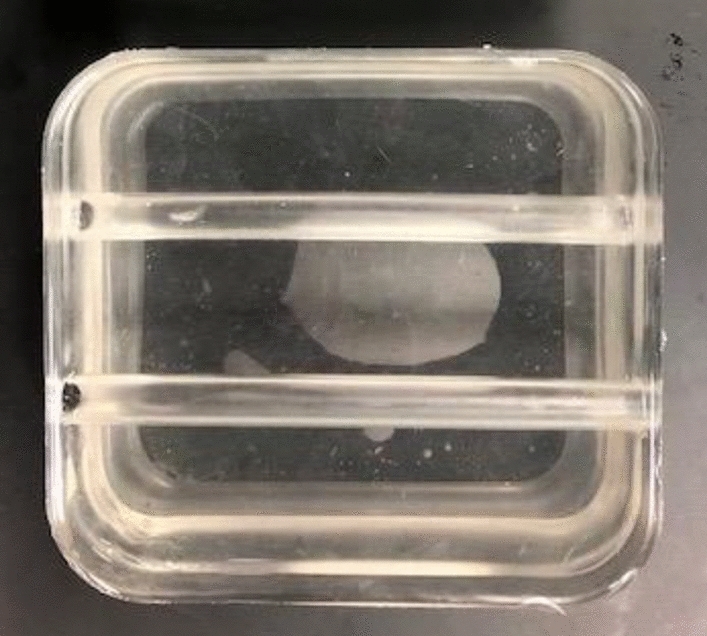
Simple tissue-mimicking phantom used for Vector Flow Imaging (VFI) and Blood Speckle Imaging (BSI) to demonstrate flow visualization.

A simple phantom containing two straight, hollow conduits was created using Gel 0 (Fig. 5). This phantom was created in order to have a predictable flow pattern and evaluate the efficacy of ultrasound-based flow imaging methods for flow visualization of the tissue-mimicking phantom. Two angle-independent, ultrasound-based imaging modalities that provide detailed vector flow visualization were used: Vector Flow Imaging (VFI) and Blood Speckle Imaging (BSI). VFI uses transverse oscillation to estimate both the axial and transverse velocity components whereas BSI utilizes high-frame rate imaging and speckle tracking of blood cells to measure and produce a velocity field.^13^, ^16^, ^28^, ^29^, ^42^ Prior to imaging, the phantom was connected to the aforementioned flow loop set-up to provide continuous flow. The fluid consisted of distilled water with cornstarch for VFI. In order to illicit a signal similar to that of blood cells for the speckle tracking, a blood-mimicking fluid (BMF) containing nylon particles was used for BSI to mimic the acoustic scatter and viscosity of human blood with *ρ* = 1037 kg/m^3^ and *μ* = 4.1 mP s.^23^, ^43^, ^47^ VFI was performed using a bk5000 system with built-in VFI (BK Medical, Peabody, MA) and 5 MHz linear probe (Linear Array 8L2, BK Medical) with and without vector arrows overlaid and at two different reference velocities: 66.7 cm/s and 261.9 cm/s. For BSI, a Vivid E95 with BSI software (GE Vingmed Ultrasound, Horten, Norway) and 6 MHz phased array probe (6S, GE Healthcare, Milwaukee, WI) were used to obtain images at a reference velocity of 23 cm/s with and without particles displaying flow trajectory.

### Statistical Analysis

One-Way ANOVA was performed on datasets from mechanical testing, followed by Tukey HSD posthoc, using JMP (SAS, Cary, NC) with *p* < 0.05 for significance. All values are reported in terms of mean ± standard deviation unless otherwise specified.

## Results

### Phantom Fabrication

**Figure 6:**
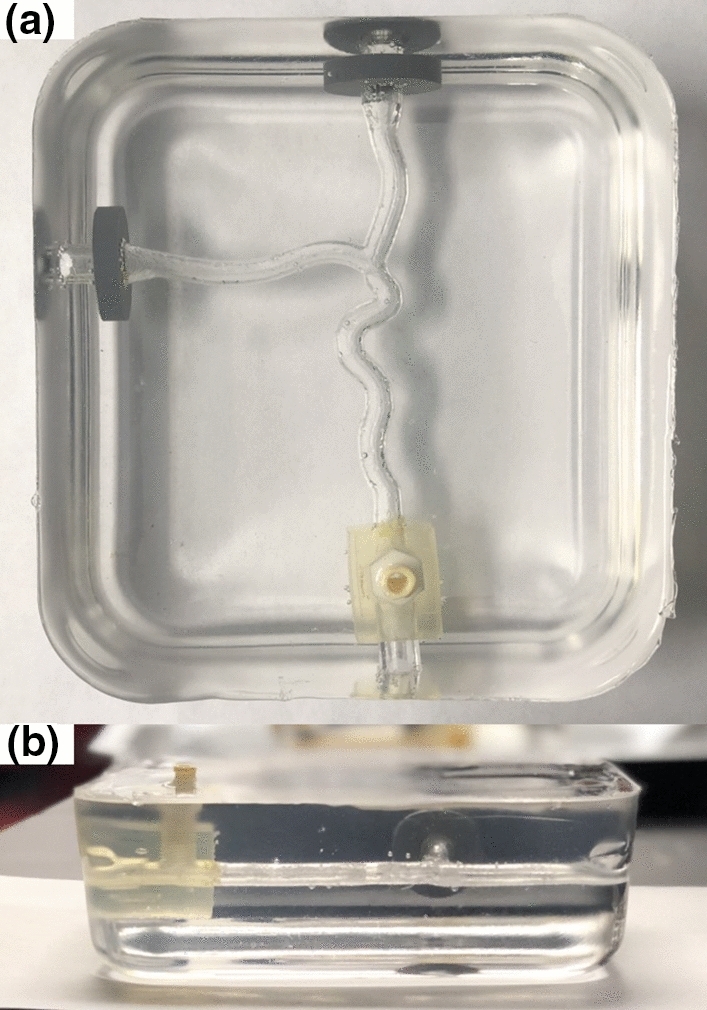
Completed phantom viewed from the (a) top and (b) side. The conduit within the gel corresponding to the bifurcated artery model can be seen and practically no bubbles are present.

The completed bifurcated artery phantom is presented in Fig. 6. The wall-less phantom is comprised of a gel block with a conduit of the desired geometry and the three embedded anchors, materials costing approximately $75 to fabricate. No bubbles of any practical importance are present. A small amount of bubble formation occurred around the anchors, which does not impact the actual functionality of the phantom. The gel is transparent with good clarity, and the conduit can be easily identified.

**Figure 7:**
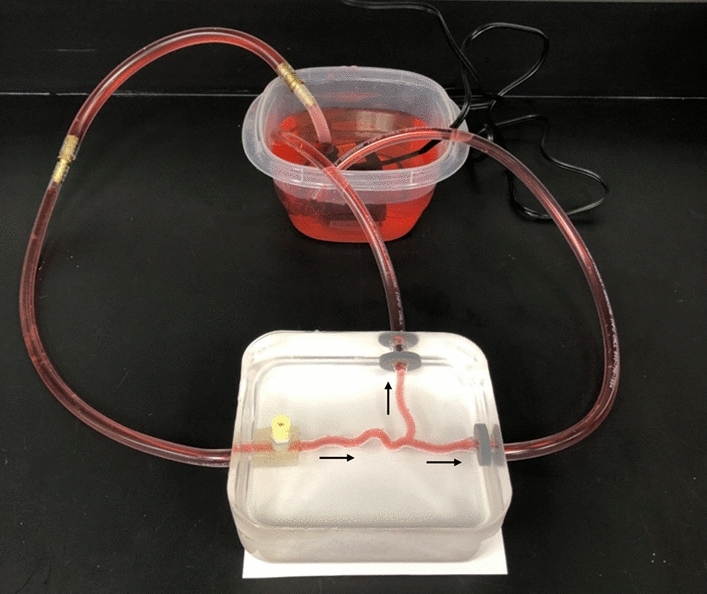
Continuous flow through the phantom is demonstrated with direction depicted by the arrows.

The anchors within the phantom allowed for secure attachment of tubing to provide fluid flow to the phantom. The presence of the Luer anchor also serves as an additional inlet/outlet to aid in the dissolution of the insert and offers the ability to attach accessories such as pressure catheters or a flow meter, which may be desired in various flow applications. Continuous flow through the phantom was demonstrated as seen by the red fluid in Fig. 7.

### Mechanical Testing

**Figure 8:**
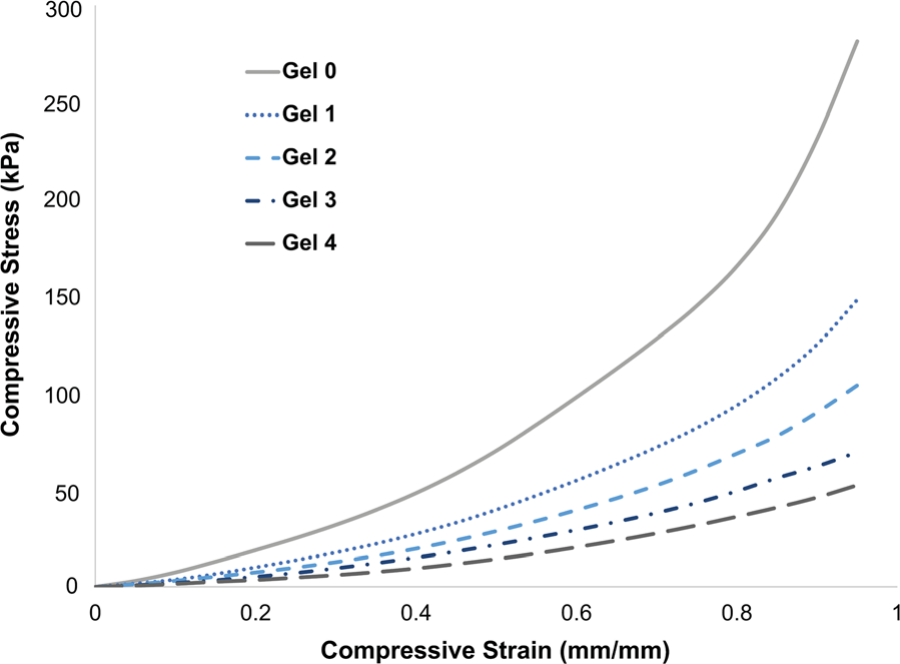
Average compressive stress–strain curves obtained from uniaxial compression testing for the five gel types.

Compressive stress–strain curves from uniaxial compression testing were obtained and used to generate an average compressive stress–strain curve for each of the five gel types (Fig. 8). The average Young’s moduli of the gels ranged from 17 to 92 kPa for 0 to 25% strain and 57 to 250 kPa for 25 to 75% strain, decreasing with increasing gel number (i.e., from Gel 0 to Gel 4) in both strain ranges (Table 1). A significant difference was observed between the Young’s moduli of all gel types at 25–75% strain (*p* < 0.10 between Gel 3 and Gel 4 and *p* < 0.05 between all other groups). Additionally, a significant difference was observed at 0–25% strain for all gel types (*p* < 0.05); however, there was no significant difference between Gel 2 and Gel 3 (*p* = 0.0687) or Gel 3 and Gel 4 (*p* = 0.24).

**Table 1:** Young’s moduli of gel types as determined from uniaxial compression testing and analysis of the 0–25% and 25–75% strain ranges

	0–25% strain		25–75% strain	
	*E* (kPa)	*R*^2^	*E* (kPa)	*R*^2^
Gel 0	92.02 ± 16	0.97	250.07 ± 23	0.98
Gel 1	49.05 ± 5	0.96	141.13 ± 15	0.99
Gel 2	36.57 ± 7	0.98	102.88 ± 13	0.98
Gel 3	25.55 ± 5	0.97	73.07 ± 6	0.99
Gel 4	17.02 ± 7	0.91	56.57 ± 9	0.97

The average *R*^2^ values from the linear fit of all gel types were 0.96 and 0.98 for 0–25% and 25–75% strain ranges, respectively (Table 1). While 0–25% strain exhibited slightly decreased linearity compared to 25–75% strain, these values indicate a good fit of the linear stress–strain relationship used to characterize the elastic properties in the analyzed ranges. All gel samples were successfully compressed to 95% strain; however, some samples began tearing beyond 75% strain with no consistency in which gel types did so. The tearing did not noticeably affect the stress–strain curves, as seen in Fig. 8. In other words, the tearing did not produce a characteristic failure region.

### Flow Visualization

**Figure 9:**
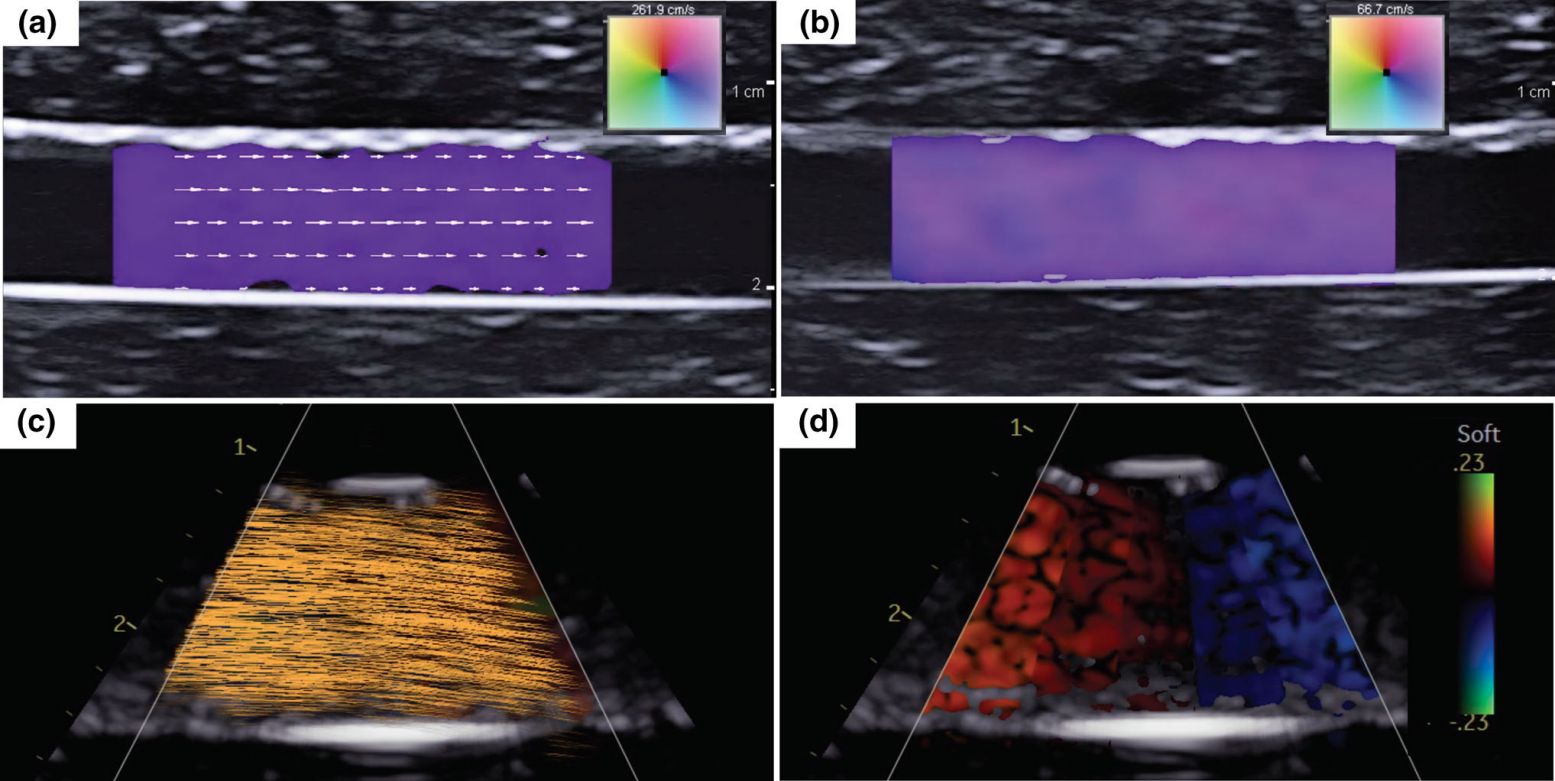
Flow visualization demonstrating unidirectional flow through the phantom using Vector Flow Imaging (VFI) with distilled water and cornstarch (a–b) and Blood Speckle Imaging (BSI) with a blood-mimicking fluid (BMF) (c–d). (a) Arrows overlaid, *v*_ref_ = 261.9 cm/s; (b) No arrows, *v*_ref_ = 66.7 cm/s; (c) Particles depicting flow trajectory; (d) Speckle pattern, *v*_ref_ = 23 cm/s. *v*_ref_ = reference velocity.

VFI and BSI were successfully performed with the phantom to demonstrate use with ultrasound-based imaging modalities and provided visualization of unidirectional flow within the phantom conduit as seen in Fig. 9. Figures 9a and 9b depict varying VFI settings, specifically the overlay of arrows and reference velocity, with consistent experimental flow conditions. The flow trajectory via the display of particles and speckle pattern from BSI are shown in Figs. 9c and 9d, respectively. From the single purple color seen in Fig. 9a, it appears as if a uniform velocity-field is present as a result of the reference velocity being high (261.9 cm/s) relative to the measured velocity through the phantom. The presence and magnitude of the arrows in Fig. 9a allow for slight differences in velocity throughout the flow field in the direction along the flow axis to be seen and demonstrate that laminar flow was not fully developed. This was likely due to the tubing being inserted into the phantom without anchors that led to expansion resulting from a mismatch between the tubing inner diameter and conduit, highlighting the importance of the interaction and precise diameter match between the tubing and phantom on flow. This flow regime is further confirmed by Fig. 9b where no arrows are overlaid but the reference velocity is lower (66.7 cm/s), making apparent the differing magnitudes in the flow field by the wider range of colors observed.

Flow visualization was achieved with BSI by using the BMF, as seen in Figs. 9c and 9d, which yields the first reported use of BSI on an *in vitro* flow phantom. The particles displaying the trajectory of flow in Fig. 9c also confirm unidirectional flow and the absence of fully developed laminar flow. This flow is also observed in Fig. 9d where the speckle pattern changes from red to blue as the direction in reference to the transducer, which is perpendicular to the flow, changes. With both VFI and BSI, the conduit and gel were distinguishable from one another by a difference in contrast on the image. Additionally, a distinct line was seen at the conduit/gel interface of the phantom.

## Discussion

We have developed a methodology for fabricating custom wall-less flow phantoms from clear tissue-mimicking gel using 3D printed PVA inserts, determined the Young’s moduli of the available gel types, and demonstrated flow visualization of the phantom with advanced ultrasound techniques. This method is low-cost relative to commercial phantoms that often cost more than $1,000 and comparable to other techniques using silicone, which have been reported to cost between $69–250 per phantom.^21^, ^48^ Recently, 3D printing of elastic materials with various mechanical properties has become feasible but cost is again a major limitation. These materials are most commonly and accurately printed using SLA or inkjet printers which present a significant upfront cost ranging from thousands of dollars for SLA to hundreds of thousands of dollars for inkjet.^12^ The presented methodology utilizes extrusion printing, also known as fused deposition modeling or fused filament fabrication, which has a much lower 3D printer cost of hundreds to a couple of thousand dollars.^46^ Furthermore, the material filaments are inexpensive compared to the resin cartridges required for SLA and inkjet printers. If cost is not a consideration, then 3D printing with an elastic material would likely be the easiest method to create a custom tissue-mimicking phantom. However, that is most often not the case and therefore this more cost-effective option is desirable.

The fabrication method allows for custom geometries limited only by what can be drawn using CAD and subsequently 3D printed. It also negates the need for harsh solvents required to dissolve other 3D printable materials and consists of minimal steps compared to alternative methods. Other materials often require preparation to manipulate or activate its properties while some even need to be made from scratch using a well-established protocol or “recipe”.^11^, ^25^ In contrast, the tissue-mimicking gel comes ready-to-use and the availability of multiple stiffnesses negates the need for additional steps to achieve the desired mechanical properties. Previous studies utilizing methods such as multi-stage investment casting are laborious with a series of casts, become more difficult with increasing geometry complexity, and some materials used must undergo several freeze–thaw cycles.^2^, ^23^ Aside from initiation and termination of each step, the developed methodology for phantom fabrication is relatively hands-off and demands less labor intensity. The ability of the gel to be reused by remelting makes it particularly user friendly and cost-efficient, as this allows for the correction of errors or imperfections, design adjustments, and potentially destructive events such as holes and tears that may be either intentional or unintentional.

The print settings ensured consistent and reliable PVA inserts but are not optimized for a high production setting. The steaming of the inserts prior to embedment resulted in a decrease of bubbles formed within the gel by creating a smooth surface which entrains significantly less air. Qualitatively, this minimized the surface roughness of the insert, which in turn minimizes the surface roughness of the phantom conduit. This is particularly important in flow applications in order to minimize minor losses within the phantom. Given the application, surface roughness measurements of the inserts/conduit may be warranted which can be done using a variety of methods such as a profilometer, phase tracking, confocal laser scanning microscopy, and optical coherence tomography.^10^, ^57^ As it has been demonstrated, the phantoms had excellent clarity. This is an important additional advantage compared to other materials that allows for ease of visual tests and may be particularly useful in tracking expansion of the gel via camera or for flow analysis using Particle Image Velocimetry (PIV) in mechanical environments that optimally mimics human tissue. The gel can also be supplied in an opaque color from the manufacturer with various skin tones available.

While no bubbles of practical importance were present, the criteria for this threshold is application specific. Flow phantoms should be free of bubbles against the insert/cavity as these are likely to affect flow conditions, whereas a few bubbles in the periphery of the phantom could be tolerable. If optical measurement methods are employed, such as PIV, or visual access to the flow field is important, care should be taken to ensure no bubbles are present in the optical path between the light source, cavity, or detector. Similar considerations would be necessary for ultrasound-based imaging applications. In our experience, any bubbles present were consistently < 1 mm in diameter. Preliminary work with a vacuum oven, which simultaneously draws a vacuum and heats via radiant heat, has demonstrated the ability to further reduce bubbles but the insert wall thickness is an important consideration to ensure the vacuum does not induce diffusion of gel into the void. This may be eliminated by sealing the void from the vacuum, which was not part of the testing since it was possible to remove bubbles to a satisfactory level for the current use without using a vacuum or vacuum oven. In applications that do not require any visual, optical, or imaging access to the flow field, bubble extraction is less critical. Examples of such applications are training phantoms for needle puncture or device catheter advancement through vessel structures.

It was observed that if left in the oven for an extended period of time (approximately > 2.5 h), the gel was capable of diffusing through thin sections of the PVA insert, thus filling the channel. This resulted in difficulty establishing flow through the phantom for dissolution of the insert and the need for manually removing pieces of gel inside. The possibility of this occurring can be minimized by ensuring an adequate balance of insert wall thickness to channel size and checking periodically that no gel is present within the channel while the phantom is in the oven. All steps within the fabrication process are dependent on the size of the overall phantom and specific geometry. In particular, the time taken to melt and debubble the gel is based to the amount of gel used and dissolution of the insert on the amount of PVA (i.e. wall thickness). In the case of the bifurcated artery phantom presented, the entire fabrication process is concluded to take between 12.5 and 33.5 h depending on the length of time allowed for cooling. The fabrication time for other methods is not often reported but is estimated to range between 1 and 10 days depending on the material, which the method proposed here falls on the lower end of.^21^

The Young’s moduli of the different gels varied significantly and exhibited a decrease with increasing gel type number in both strain ranges analyzed, spanning a range of stiffnesses. This demonstrates a significant advantage of these gels as they can be matched to a variety of tissues involving flow. Specifically, it has been shown that Young’s moduli values range from 8 to 500 kPa for cardiac muscle and 200–6000 kPa for blood vessels.^17^, ^18^, ^22^, ^27^, ^32^, ^33^ Furthermore, the gels have potential for use in phantoms mimicking other tissue types including breast tissue (0.5–66 kPa), skin (1.5–1000 kPa), and skeletal muscle (5–170 kPa), and given their mechanical properties may have particular usefulness in elastography applications.^6^, ^14^, ^20^, ^22^, ^30^, ^34^, ^36^, ^50^ The Young’s moduli of various tissues are given in Table 2 and can be compared to those of the available gel types (Table 1) to ensure the appropriate gel is chosen to mimic a particular tissue. However, it is to be noted that a wide range of values have been reported for the Young’s modulus of biological tissues and are highly dependent on testing methods and conditions.^1^, ^14^, ^31^ Therefore, it is important to take into consideration the specific application the phantom will be used for and the forces (i.e., direction) and/or strains it may undergo to determine the appropriate Young’s modulus of the tissue to be mimicked.

**Table 2:** Young’s moduli of biological tissues that have the ability to be mimicked by the available gel types

Tissue	Young’s modulus (kPa)
Breast tissue	0.5–66^20^, ^32^
Skin	60–1000^1^, ^10^, ^14^, ^50^
Skeletal muscle	5–170^10^, ^34^, ^36^
Cardiac muscle	8–500^10^, ^18^, ^22^, ^33^
Blood vessels	200–6000^17^, ^27^

**Figure 10:**
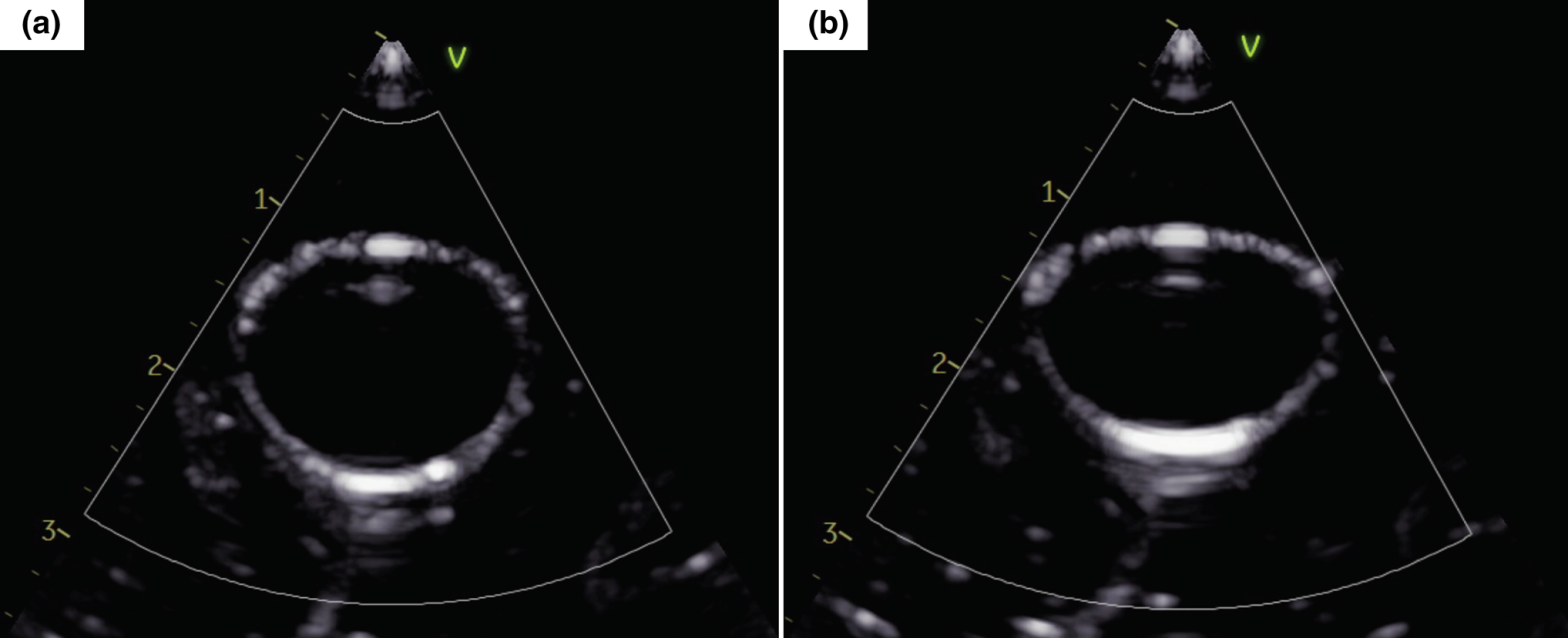
Preliminary example of the tissue-mimicking phantom (a) with and (b) without compressive strain induced by force exerted on the phantom via an ultrasound transducer to demonstrate the potential for use in strain elastography.

The gels exhibited an overall non-linear elastic compressive stress–strain curve and did not present a noticeable failure region even though tearing was experienced in some gels when approaching 95% strain. The shape of these curves is characteristic of many soft tissues, including those in and surrounding the cardiovascular system, where there are distinct low and high strain regions with low and high elastic moduli, respectively.^17^, ^56^ The fact that the gels exhibit this behavior further demonstrates their mimicry of human tissue and can be advantageous to numerous applications, including ultrasound elastography. Strain elastography is particularly relevant to this work due to the Young’s moduli being evaluated in compression. A preliminary example of the behavior of the gel under compressive strain induced by the exertion of force via a transducer is given in Fig. 10 and demonstrates its potential for this use.

Continuous flow through the phantom was demonstrated and visualization confirmed using VFI and BSI. Images confirmed the ability to non-visually detect flow through the phantom and distinguish differences between conduit and gel. This exhibits the ability to utilize these phantoms for applications where flow may be quantified using methods such as ultrasound-based imaging modalities or PIV. However, flow was only qualitatively visualized in this work due to the main focus being on the tissue-mimicking phantoms and therefore the velocity must be inferred from the displayed color square/bar. Future studies will focus on flow quantification using such modalities on these phantoms. It is sometimes desired that phantoms also have acoustic and optical properties that match the specific tissue to be mimicked, which can be particularly important for use with certain imaging modalities and applications such as quality control.^4^, ^5^, ^41^ Multiple studies have reported the use of various additives and scattering materials to tissue-mimicking materials in order to control the acoustic and optical properties.^8^, ^37^, ^39^ A study by Alves *et al*. utilizing the same tissue-mimicking gel combined with mineral oil and cellulose powder reported acoustic properties within the range of cardiac tissue and standards for phantoms.^2^ Therefore, it is believed that the gels can exhibit the desired acoustic and optical properties whether as-is or through tuning, which would be an advantage over certain materials like silicone that may in some configurations sufficiently mimic mechanical properties but do not exhibit ideal acoustic and optical properties. For this, further studies are necessary.^7^, ^35^, ^62^ For flow phantoms, the fluid properties are particularly important to consider, as demonstrated by the need of a BMF for successful flow visualization with BSI. Numerous options of BMFs have been determined that mimic the density, viscosity, speed of sound, and backscatter of blood as well as the refractive index of various tissue-mimicking materials.^3^, ^8^, ^23^, ^25^, ^43^, ^44^, ^61^ The most suitable fluid should be chosen based on the needs presented by a given application. With the combination of flow and the mechanical behavior of the gel, these phantoms have great potential for use alongside Computational Fluid Dynamics methods such as Immersed Boundary or Fluid–Structure Interaction.

There are additional limitations of this work that ongoing and future studies will aim to address. Although numerous studies have demonstrated the usefulness of wall-less flow phantoms, particularly with ultrasound-based methods, there are applications where this may not sufficiently mimic *in vivo* tissue behavior. Therefore, future work will expand upon current methods to fabricate custom walled flow phantoms using this tissue-mimicking gel that exhibit variable gel thickness of a desired geometry. In general, walled flow phantoms present their own unique set of challenges such as increased difficulty in creating complex geometries and consistent wall thickness along with increased cost.^7^, ^40^, ^59^ Additionally, this study utilized continuous flow whereas pulsatile flow is often desired for cardiovascular flow phantoms. Preliminary experimentation has qualitatively shown realistic mechanical reactions of the gel phantoms in response to pulsatile flow. Future aspects of this research will characterize this response to pulsatile flow by quantifying cavity expansion and evaluating the effects of gel thickness on mechanical behavior. This may be important in using the phantoms for pulsatile flow applications, since compliance is known to be inversely proportional to wall thickness.^59^ Further mechanical testing may be warranted, such as tensile testing and dynamic mechanical analysis to elucidate the elastic and viscous responses, with the extension of methods where tension and oscillatory forces become more prevalent.^8^

## Conclusions

A method of embedding and removing 3D printed PVA inserts in tissue-mimicking gel to yield custom wall-less flow phantoms with physiologically relevant mechanical properties of various tissues has been demonstrated. The materials and overall fabrication process are low-cost and user friendly. Flow visualization of the phantom was demonstrated with advanced ultrasound flow imaging techniques and offers the prospect of flow quantification using a variety of methods. This is the first reported use of BSI on an *in vitro* flow phantom, which requires use of a BMF. In addition, we have identified various tissues and applications that may be suitable for use with these tissue-mimicking phantoms.

## Data Availability

The data that support the findings of this research are openly available in the Mendeley Data database at http://dx.doi.org/10.17632/xprsymw64w.1.

## Abbreviations

BMF: Blood-mimicking fluid
BSI: Blood speckle imaging
CA: Cyanoacrylate
PIV: Particle image velocimetry
PVA: Polyvinyl alcohol
SLA: Stereolithography
VFI: Vector flow imaging

## Animal Studies

No animal studies were carried out by the authors for this article.
